# Genetic Algorithm-Based Optimization for Color Point Cloud Registration

**DOI:** 10.3389/fbioe.2022.923736

**Published:** 2022-06-29

**Authors:** Dongsheng Liu, Deyan Hong, Siting Wang, Yahui Chen

**Affiliations:** ^1^ School of Computer and Information Engineering, Zhejiang Gongshang University, Hangzhou, China; ^2^ School of Management Engineering and E-commerce, Zhejiang Gongshang University, Hangzhou, China; ^3^ Zhejiang Gongshang University, Hangzhou, China

**Keywords:** genetic algorithm, point cloud, hsv, registration, optimization

## Abstract

Point cloud registration is an important technique for 3D environment map construction. Traditional point cloud registration algorithms rely on color features or geometric features, which leave problems such as color affected by environmental lighting. This article introduced a color point cloud registration algorithm optimized by a genetic algorithm, which has good robustness for different lighting environments. We extracted the HSV color data from the point cloud color information and made the HSV distribution of the tangent plane continuous, and we used the genetic algorithm to optimize the point cloud color information consistently. The Gauss–Newton method was utilized to realize the optimal registration of color point clouds for the joint error function of color and geometry. The contribution of this study was that the genetic algorithm was used to optimize HSV color information of the point cloud and was applied to the point cloud registration algorithm, which reduces the influence of illumination on color information and improves the accuracy of registration. The experimental results showed that the square error of color information saturation and lightness optimized by the genetic algorithm was reduced by 14.07% and 37.16%, respectively. The color point cloud registration algorithm in this article was reduced by 12.53% on average compared with the optimal result algorithm RMSE.

## 1 Introduction

Point cloud registration is an important technique in the fields of pose estimation, three-dimensional positioning, and computer vision. It is commonly used in simultaneous localization and mapping (SLAM), three-dimensional reconstruction, unmanned driving, and automation applications. Robots use devices such as depth cameras to make three-dimensional maps of the environment to realize the perception of the environment. Due to the limitations of shooting equipment and physical occlusion, scanning devices can only scan part of an object or scene. Therefore, the object or scene is scanned in different angles and directions to obtain the complete point cloud data. The purpose of point cloud registration is to compute the rotation and translation matrices between multiple point clouds and finally merge them into complete point cloud data.

Point cloud registration relies on geometry or color information for the moment. Douadi et al. (2006) emphasized the influence of color information on improving the registration accuracy of high point cloud for the existing problems of ICP. Tong and Ying (2018) proposed a rough matching algorithm based on texture information and point cloud curvature characteristics. Park et al. (2017) proposed a way to constrain both geometric and color information. By using the distribution of color on the tangent plane, a continuous color gradient was defined to represent the functional change of color with the location. A joint error function consisting of geometric and color errors was constructed, effectively constraining both geometric and color information. However, the color space used is the RGB color space, and then, the color information needs to be converted to gray scale information, which is easily affected by ambient light, resulting in large data fluctuations.

The aforementioned method combines color information with geometric information to improve the registration’s effectiveness, but there is inconsistency in the original color data. When the depth camera collects data, the color information collected by the depth camera changes due to the inconsistency in scene illumination and the change in the acquisition of the angle. Therefore, Ren et al. (2021) converted the RGB color space to a hue color space to improve robustness under different lighting conditions. However, saturation and brightness information is ignored.

Since the saturation and brightness of images are greatly different from hues owing to the influence of ambient lighting, it is difficult to effectively apply the saturation and brightness information to point cloud registration. With the help of previous researchers, this article was based on the study of two-dimensional image illumination uniformity; the global brightness transformation can effectively reduce the differences in brightness. The saturation feedback algorithm can effectively change the image of saturation, but this kind of algorithm relies heavily on parameter setting; setting the same parameters is not effective for all situations. The genetic algorithm has the abilities of fast random search, simple process, easy to integrate with other algorithms, can effectively haphazard search for appropriate parameters, and reduce the impact of the environment on color information.

This article proposed a color point cloud registration algorithm optimized by a genetic algorithm to solve the aforementioned problems in point cloud registration. In this algorithm, the global luminance of color information is logarithmic transformation, and the HSV (hexcone model) color information is optimized by using a genetic algorithm. According to the normal vector and the surface curvature of the point, the matching correlation points of the point cloud are calculated. Finally, the error function composed of geometric error and color error is constructed. Experiments showed that the algorithm proposed in this article can also realize accurate point cloud registration with the help of color information and geometric information in the case of geometric degradation and complex scenes, which proves that the algorithm can improve the accuracy of the color point cloud registration.

The contributions of this article are as follows:

1) Aiming at the fact that the saturation and brightness of the HSV color space are greatly affected by illumination, the global brightness transformation and saturation feedback algorithm of the 2D image is applied to 3D point cloud registration.
2) We constructed a fitness index to evaluate saturation information, optimized the consistency of point cloud color information by using a genetic algorithm, and effectively reduced the influence of environmental illumination on point cloud registration.

The remaining article is organized as follows. The *Related Work* section discusses some work of the point cloud registration in recent years, and the *Algorithm Implementation* section shows the specific algorithm steps. The *Experimental Result and Analysis* section shows the experimental results and analysis, and the *Conclusion* summarizes the article and the future research direction.

## 2 Related Work

Point cloud registration has been extensively investigated in history. The point cloud registration method mainly includes an optimization-based registration algorithm, feature extraction-based registration algorithm, and end-to-end registration algorithm (Huang et al., 2021). The most classical point cloud registration algorithm based on optimization is the ICP algorithm (Besl and McKay, 1992). ICP achieves registration of target point clouds by finding the nearest point neighbor in space as a matching pair and calculating a three-dimensional rigid transformation using matching points. However, the challenging assignment corresponding to the nearest point based on spatial distance is sensitive to the initial rigid transformation and outliers. This often leads to ICP convergence to the wrong local minimum. To solve the problems being completed in ICP, many studies have put forward improvement schemes for the shortcomings of the original ICP. Rusinkiewicz and Levoy (2001) considered the distance between the vertex and the target vertex and proposed a point-to-surface ICP algorithm, which made the iteration process tough to fall into local optimum. Grant et al. (2019) proposed a surface-to-surface ICP algorithm, which not only considered the local structure of the target point cloud but also the local structure of the source point cloud. Segal et al. (2009) proposed a generalized ICP (GICP). It combines the point-to-point and surface-to-surface strategies to improve accuracy and robustness. Serafin and Grisetti (2015) added the curvature constraints of the normal vector and point cloud to point cloud registration and considered the distance from the point to the tangent and the angle difference of the normal vector, which improved the accuracy of the registration. But these algorithms only constrain the geometrical information of the point cloud and do not make effective use of color information.

With the popularization of 3D lidar and depth cameras, depth maps and color map data can be obtained simultaneously with corresponding equipment. Therefore, many articles have been proposed to improve the accuracy of the point cloud registration by combining depth map and color map data. Traditional point cloud registration methods based on color information extend the color information vector from three-dimensional to a higher dimension (Besl and McKay, 1992; Men et al., 2011; Korn et al., 2014; Jia et al., 2016). Jia et al. (2016) improved homogeneity and registration accuracy by converting the RGB color space into the LAB color space and combining the NICP algorithm. Ren et al. (2021) reduced the impact of ambient brightness changes by converting the RGB color space into hue color space.

In addition, some research practices construct a point cloud registration algorithm based on feature extraction. Hu et al. (2021) used the scale-invariant feature transform (SIFT) algorithm to extract scale-invariant features from the 2D gray image, which improves the matching accuracy. Ran and Xu (2020) proposed a point cloud registration method that integrates sift and geometric features. The feature points are produced by SIFT, and incorrect matching points are evaluated by curvature.

In addition to the traditional point cloud registration algorithm mentioned previously, with the progress of deep learning in recent years, many researchers have proposed the registration algorithm based on deep learning (Guo et al., 2020; Kurobe et al., 2020; Huang et al., 2021). The current depth-based registration algorithms are mainly divided into feature-based learning point cloud registration algorithms and end-to-end–based point cloud registration algorithms. The feature-based point cloud registration algorithm mainly evaluates features through input point cloud and deep network, then solves the rotation translation matrix, and outputs the results. In recent years, DCP (deep closest point) (Wang and Solomon, 2019), RPMNet (Yew and Lee, 2020), AlignNet (Groß et al., 2019), and other models have been developed. The main approach of the point cloud registration algorithm based on end-to-end is to input two pieces of the point cloud. Generally, the neural network fitting regression model is used to estimate the rotation translation matrix or to combine the neural network with optimization. In recent years, DeepGMR (Yuan et al., 2020) and 3D RegNet (Pais et al., 2020) have been used as models. The main advantage of a registration algorithm based on deep learning is that it can combine the advantages of a traditional mathematical theory and neural network well. But calculating the in-depth learning model at the same time requires a lot of training data and often only works well for scenarios contained in the training set. When facing untrained scenarios, the accuracy will be greatly reduced. In addition, there is a high requirement for training effort (Guo et al., 2020).

## 3 Algorithm Implementation

**FIGURE 1:**
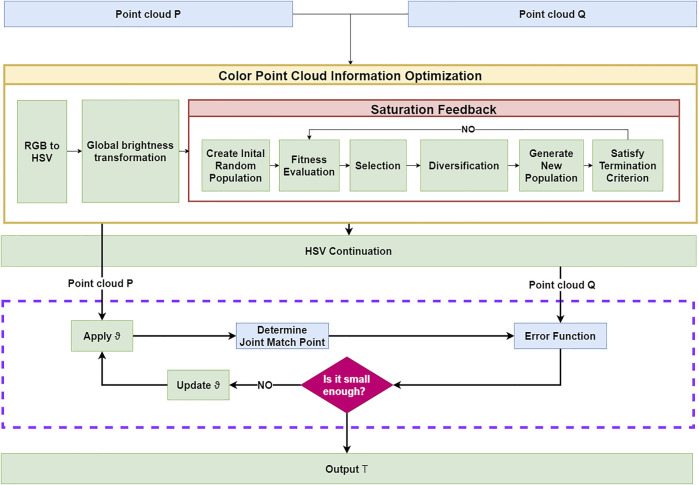
Algorithm process.

In this article, a color point cloud registration algorithm based on genetic image enhancement and geometric features is designed. The algorithm flow is shown in Figure 1. The algorithm consists of four steps. Step 1: the global luminance logarithmic transformation should be performed on the color information, and then, the HSV color information should be optimized based on a genetic algorithm to calculate the color information gradient. Step 2: the association points of the joint image features and geometric features query point cloud should be extracted, and the association points that do not meet the conditions should be filtered. Step 3: the error function combining the geometric error and color error should be constructed. Step 4: The Gauss–Newton method should be used to solve the point cloud iteratively.

### 3.1 Color Point Cloud Information Optimization

In general, the original image acquired by the depth camera is a RGB image. The RGB color space is the most frequently used color space mode. It composes of three primary colors: red, green, and blue. The range of red, green, and blue is between 0 and 255, which is widely used in the field of computer vision. However, the uniformity of the RGB color space is insignificant; the color difference cannot be judged directly by spatial distance, and the brightness cannot be handled well. At the same time, due to the influence of ambient light brightness, images from different angles lead to coloring deviation.

To solve the aforementioned problem, this article puts forward after a genetic algorithm to optimize the HSV color information with the introduction of point cloud registration methods; this article is first to study the global image brightness logarithmic transformation and complete the dark areas in the image enhancement and dynamic range compression. To further improve the optimization of HSV color information, this article used a genetic algorithm to dynamically calculate the saturation optimization coefficient and to improve the stability of saturation under different lighting conditions.

The global logarithmic brightness transformation is applicable to the original image. The global logarithmic brightness transformation formula is as follows:

$$V^{\prime }\left(x,y\right)=\ln\left(V\left(x,y\right)+1\right),$$ (1)

where *V*′(*x*, *y*) is the logarithmic brightness, and *V*(*x*, *y*) is the original brightness at the point (*x*, *y*). To reduce the influence of illumination on brightness, this article first performed a global logarithmic brightness transformation on the original image. Setting the red, green, and blue channels in each original RGB image as *R*_*e*_, *G*_*e*_, *B*_*e*_, and *C*_max_ as the largest of the three channels, the global logarithmic brightness transformation formula is as follows:

$${R_{e}}^{\prime }=R_{e}/255,{G_{e}}^{\prime }=G_{e}/255,{B_{e}}^{\prime }=B_{e}/255;$$ (2)

$$C_{\max}=\max\left(R_{e}^{\prime },G_{e}^{\prime },B_{e}^{\prime }\right),C_{\min}=\min\left(R_{e}^{\prime },G_{e}^{\prime },B_{e}^{\prime }\right),\Delta =C_{\max}-C_{\min};$$ (3)

where $R_{e}^{\prime }$ , $G_{e}^{\prime }$ , and $B_{e}^{\prime }$ , respectively, represent the normalized results of *R*_*e*_, *G*_*e*_, and *B*_*e*_ in corresponding places; *C*_min_ is the minimum value of *R*′, *G*′, and *B*′; and Δ is the difference between the maximum value and minimum value of $R_{e}^{\prime }$ , $G_{e}^{\prime }$ , and $B_{e}^{\prime }$ .

$$H=\left\{\begin{array}{cc} \Delta =0 & 0{}^{\circ};\\ \frac{60{}^{\circ}\times \left(\frac{G_{e}^{\prime }-B_{e}^{\prime }}{\Delta }\;\mathrm{mod}\;6\right)}{360} & ,C_{\max}^{\prime }=R_{e}^{\prime };\\ \frac{60{}^{\circ}\times \left(\frac{B_{e}^{\prime }-R_{e}^{\prime }}{\Delta }+2\right)}{360} & ,C_{\max}^{\prime }=G_{e}^{\prime };\\ \frac{60{}^{\circ}\times \left(\frac{R_{e}^{\prime }-G_{e}^{\prime }}{\Delta }+4\right)}{360} & ,C_{\max}^{\prime }=B_{e}^{\prime }; \end{array}\right.$$ (4)

$$S=\left\{\begin{array}{cc} 0 & ,C_{\max}=0;\\ \frac{\Delta }{C_{\max}} & ,C_{\max}\neq 0; \end{array}\right.$$ (5)

$$V=C_{\max}.$$ (6)

$V\in \left[0,1\right],S\in \left[0,1\right],andH\in \left[0,1\right)$ . because the global logarithmic brightness transformation only enhances the brightness while the saturation remains essentially the same. Therefore, to optimize saturation effectively, this article is based on a genetic algorithm and feedback saturation enhancement algorithm to enhance the saturation layer (Strickland et al., 1987).

The genetic algorithm simulates the survival of the fittest in nature and uses selection, crossover, and variation to solve a satisfactory solution. According to the saturation feedback formula, this article modified it as follows:

$$S^{*}\left(x,y\right)=k_{s}\left[S^{\prime }\left(x,y\right)-{\bar{S}}^{\prime }\left(x,y\right)\right]+k_{v}\left[V^{\prime }\left(x,y\right)-{\bar{V}}^{\prime }\left(x,y\right)\right]+S\left(x,y\right),$$ (7)

where *S*∗(*x*, *y*) is the enhanced saturation value, *S*′(*x*, *y*) = 1 − *S* (*x*, *y*), and *S*(*x*, *y*) is the saturation at the point (*x*, *y*); ${\bar{S}}^{\prime }\left(x,y\right)$ is the local saturation mean at the point (*x*, *y*), *V*′(*x*, *y*) = 1 − *V*(*x*, *y*), and ${\bar{V}}^{\prime }\left(x,y\right)$ is the local saturation mean at the point (*x*, *y*). Because the coefficients of *k*_*s*_ and *k*_*v*_ are very important for saturation processing, the manual setting method cannot effectively meet the actual need. For this reason, this article used a genetic algorithm to calculate the saturation coefficient dynamically so that it can adjust the values of *k*_*s*_ and *k*_*v*_ adaptively.

The genetic algorithm in this article is divided into four steps; the specific steps are as follows:

1) Confirming the the encoding and fitness functions: float number encoding is employed in this article. *k*_*s*_ and *k*_*v*_ are the coefficients to be optimized, and their value ranges are 0–4. To test and evaluate the enhancement effect, we defined a fitness function, and it is constructed as follows:

$$f_{\mathrm{fitness}}=\exp\left({\left[\frac{1}{M\times N}\overset{M}{\underset{x=1}{\sum }}\overset{N}{\underset{y=1}{\sum }}S\left(x,y\right)\right]}^{2}-\frac{1}{M\times N}\overset{M}{\underset{x=1}{\sum }}\overset{N}{\underset{y=1}{\sum }}S{\left(x,y\right)}^{2}\right),$$ (8)

For an image of *M* × *N* size, the smaller the ***f***_*fitness*_ value, the larger is the average change of image saturation.

2) Identifying the selection strategy and the genetic operator: to ensure the optimal individuals can be obtained, this article used the sorting selection strategy, as shown in Eq. 9. In addition, in order to maintain the diversity of the group, it is not permitted to choose the same parent.

$$P\left(S^{*}{\left(x,y\right)}_{i}\right)=\frac{f_{\mathrm{fitness}}\left(S^{*}{\left(x,y\right)}_{i}\right)}{\sum_{j=1}^{N}f_{\mathrm{fitness}}\left(S^{*}{\left(x,y\right)}_{j}\right)}.$$ (9)

3) Determination of the control parameters of the genetic algorithm: it mainly includes population size, variation rate, and crossover rate. If the size of the population is too small and lacks diversity, it can easily lead to regional convergence. A large population size makes the algorithm converge slowly, which influences the processing speed. Therefore, the size of the population should be large enough. In the experiment, we set the population size at 30 and the crossover rate and variation rate at 0.9 and 0.05, respectively.
4) Determination of downtime criteria: the calculation is aborted based on the maximum number of iterations and the rate of change between the average fitness value of the current population and that of the previous generation. The maximum number of iterations is set to 100, and the change rate of the average fitness is 0.2%. When the number of iterations is greater than 100 or the change rate of average fitness is less than 0.2%, the calculation is aborted.

In order to optimize the error of color information, it is necessary to obtain the corresponding color information gradient. Therefore, *H*(**p**) needs to be converted into the continuous color function *H*_**p**_(*v*), where *v* is the tangent plane vector, and *N*_**p**_ is the neighborhood of **p**; **p**′ is a point in *N*_**p**_, and ***n***_**p**_ is the normal of the point **p**, making v* ***n***_**p**_ = 0. We assumed that *H*_**p**_(*v*) is a continuous function and represented the distribution of HSV on the tangent plane; then it can define *H*_**p**_(*v*) as follows:

$$H_{p}\left(v\right)\approx H\left(p\right)+d_{p}^{T}v,p\in P.$$ (10)

For each point **p**, *f*(*s*) is the plane function projected by point P to the section plane:

$$f\left(s\right)=s-n_{p}{\left(s-p\right)}^{⊤}n_{p}.$$ (11)

The least square fitting objective of *d*_**p**_ is as follows:

$$\begin{array}{cc} L\left(d_{p}\right) & =\underset{p^{\prime }\in N_{p}}{\sum }{\left(H_{p}\left(f\left(p^{\prime }\right)-p\right)-H\left(p^{\prime }\right)\right)}^{2}\approx \underset{p^{\prime }\in N_{p}}{\sum }{\left(H\left(p\right)+d_{p}^{⊤}\left(f\left(p^{\prime }\right)-p\right)-H\left(p^{\prime }\right)\right)}^{2} \end{array}.$$ (12)

There is a constraint that requires $d_{p}^{T}n_{p}=0$ , which is solved by using the Lagrange multiplier method in this article.

### 3.2 Point Cloud-Associated Points

The traditional way to find the associated points of the point cloud usually only depends on the geometric information to find the matching points and does not make full use of the color information. Due to the scale invariance, rotation invariance, and angle invariance of sphere features, the same feature information can still be maintained at different angles and distances. At the same time, the extraction speed of orb features is significantly improved compared with SIFT (scale-invariant feature transform) and surf (speed up robot features) (Rublee et al., 2011). Therefore, in order to effectively utilize the feature and geometric information of color images, the algorithm uses the orb feature as the image feature extraction algorithm and combines the KD-tree nearest neighbor algorithm with the set of matching points found by the orb feature algorithm.

We set the orb feature point set as $K_{\text{orb }}$ and the geometric point set as $K_{kd}=\left\{\left(p_{i},q_{j}\right)\right\},p_{i}\in P,q_{j}\in Q$ feature point set.

$$K=K_{\text{orb }}\cup K_{kd}.$$ (13)

KD-Tree is used to search for ***p*** radius, and the search parameter is set at 0.04. Center $\mu_{i}^{S}$ of all points centered on ***p***_*i*_, and the covariance $\Sigma_{i}^{S}$ of the Gaussian distribution is calculated.

$$\mu_{i}^{S}=\frac{1}{\left|V_{i}\right|}\underset{p_{i}\in V_{i}}{\sum }p_{i},$$ (14)

$$\Sigma_{i}^{S}=\frac{1}{\left|V_{i}\right|}\underset{p_{i}\in V_{i}}{\sum }\left(p_{i}-\mu_{i}\right){\left(p_{i}-\mu_{i}\right)}^{T},$$ (15)

where $V_{i}$ is the set of adjacent points of point ***p***_*i*_, and *μ*_*i*_ is the center of $V_{i}$ . By matrix decomposition of $\Sigma_{i}^{S}$ , the eigenvalues *λ*_1_, *λ*_2_, and *λ*_3_ of $\Sigma_{i}^{S}$ are obtained. In this article, *σ*_i_ is used to represent the curvature of the current point to evaluate the degree of coincidence between planes. The smaller *σ*_i_ is, the flatter is the plane.

$$\sigma_{\text{i}}=\lambda_{1}/\left(\lambda_{1}+\lambda_{2}+\lambda_{3}\right).$$ (16)

Detail curvature filtering principle in the NICP algorithm is used to filter the matching points (Serafin and Grisetti, 2015).

### 3.3 Error Function

The traditional error function only considers the geometric information. When similar geometric structures occur, it is not difficult to fall into the local optimal solution. In view of the aforementioned problems, the error function set out in this article not only considers the geometric information but also the color information. *P* and *Q* are color point clouds, respectively, and *T* is the initial alignment matrix. The goal is to determine the optimal solution *T* from *P* and *Q*.

The following error functions *E*(T) are defined in this article:

$$E\left(T\right)=\left(1-\lambda \right)E_{H}\left(T\right)+\lambda E_{G}\left(T\right).$$ (17)

*E*_*H*_ (T) and *E*_*G*_ (T) correspond to the color error and geometric error, respectively. They have the following relationship:

$$q^{\text{t}}=R\left(w\right)q+t,$$ (18)

$$q^{\text{p}}=f\left(q^{t}\right),$$ (19)

where *R*(*w*) ∈ *R* ^3 × 3^ is the rotation matrix of *w*, and *t* is the displacement vector. *q* ^t^ is the new point after *q* rotation and translation transformation. *q* ^p^ is the projection of *q* ^t^ on the tangent plane; then, the HSV color error *E*_*H*_(*T*) is defined as follows:

$$E_{H}\left(T\right)=\underset{\left(p,q\right)\in K}{\sum }{\left(H_{p}\left(q^{p}\right)-H_{p}\left(q\right)\right)}^{2}.$$ (20)

The geometric error *E*_*G*_ is defined as the tangent plane distance between *q* ^t^ and *p*, ***n***_*p*_ is the normal of the point *p*; then, the geometric error is as follows:

$$E_{G}\left(\;T\right)=\underset{\left(p,q\right)\in K}{\sum }{\left({\left(q^{\text{t}}-p\right)}^{⊤}n_{p}\right)}^{2}.$$ (21)

*E*_*H*_ and *E*_*G*_ are combined to construct a complete error function:

$$E\left(T\right)=\left(1-\lambda \right)\underset{\left(p,q\right)\in K}{\sum }{\left(e_{H}^{\left(p,q\right)}\left(T\right)\right)}^{2}+\lambda \underset{\left(p,q\right)\in K}{\sum }{\left(e_{G}^{\left(p,q\right)}\left(T\right)\right)}^{2},$$ (22)

where $e_{H}^{\left(p,q\right)}$ and $e_{G}^{\left(p,q\right)}$ represent the residuals of color information and geometric information, respectively:

$$\begin{array}{c} e_{H}^{\left(p,q\right)}\left(T\right)=H_{p}\left(q^{p}\right)-H_{p}\left(q\right) \end{array}.$$ (23)

$$\begin{array}{c} e_{G}^{\left(p,q\right)}\left(T\right)={\left(q^{\text{t}}-p\right)}^{⊤}n_{\text{p}} \end{array}.$$ (24)

### 3.4 Point Cloud Registration Iteration

The Gauss–Newton method is used to solve the minimum error of *E*(*T*). In order to facilitate the algorithm calculation, we defined six-dimensional vector *ϑ* to represent *T*, and its structure is as follows:

$$\vartheta =\left(\Delta \delta ,\Delta \varepsilon ,\Delta \epsilon ,\Delta a,\Delta b,\Delta c\right).$$ (25)

Since *E*(*T*) is composed of *E*_*H*_(*T*) and *E*_*G*_(*T*), the Jacobian matrix also needs to be composed of $J_{e_{H}}$ and $J_{e_{G}}$ :

$$J_{e}=\left[\begin{array}{c} \sqrt{1-\lambda }J_{e_{H}}\\ \sqrt{\lambda }J_{e_{G}} \end{array}\right].$$ (26)

*ϑ* ^*k*^ represents the transformation vector after *k* iterations, and then, the Jacobian matrix calculation of *ϑ* ^*k*^ is as follows:

$$\begin{array}{c} J_{e_{H}}={\left.\nabla e_{H}^{\left(p,q\right)}\left(\vartheta \right)\right|}_{\vartheta =\vartheta^{k}},\\ J_{e_{G}}={\left.\nabla e_{G}^{\left(p,q\right)}\left(\vartheta \right)\right|}_{\vartheta =\vartheta^{k}} \end{array}.$$ (27)

According to the chain rule, $\nabla e_{H}^{\left(p,q\right)}\left(\vartheta \right)$ and $e_{G}^{\left(p,q\right)}\left(\vartheta \right)$ can be calculated by the following formula:

$$\begin{array}{c} \nabla e_{H}^{\left(p,q\right)}\left(\vartheta \right)=\left[\frac{\partial }{\partial \vartheta_{i}}\left(q^{p}-p\right)-H\left(q\right)\right]=\nabla H_{p}\left(f\right)J_{f}\left(s\right)J_{s}\left(\theta \right) \end{array},$$ (28)

$$\begin{array}{c} \nabla e_{G}^{\left(p,q\right)}\left(\vartheta \right)=\left[\frac{\partial }{\partial \theta_{i}}{\left(q^{t}-p\right)}^{T}n_{p}\right]=n_{p}^{T}J_{s}\left(\theta \right) \end{array},$$ (29)

where ∇*H*_**p**_(*f*) = *d*_*p*_, *J*_*f*_(*s*) is the Jacobian of *f* with respect to *s*, and *J*_*s*_(*θ*) is the Jacobian with respect to *θ*. The Gauss–Newton method is used for optimization iteration of *ϑ*, and the specific update steps are as follows:

$$\vartheta^{k+1}=\vartheta^{k}-{\left(J_{e}^{T}J_{e}\right)}^{-1}J_{e}^{T}e,$$ (30)

where $e=\left[\begin{array}{c} \sqrt{1-\sigma }e_{H}\\ \sqrt{\sigma }J_{e_{G}}e_{G} \end{array}\right]$ , when the value of error function is less than the threshold value through repeated iterative optimization of *ϑ*, the iteration is stopped, and the final result is the output.

## 4 Experimental Result and Analysis

### 4.1 Experimental Environment Configuration

We evaluated the effectiveness of the color point cloud matching algorithm based on the genetic algorithm. RawFoot (Cusano et al., 2015) and TUM datasets (Sturm et al., 2012) were used for verification, respectively. From the corn sample set in the RawFoot dataset, 64 images with different light intensities and angles are selected and used to assess color consistency. In the depth and color images, four datasets were extracted from the tums with clear 254 overlaps. It can be further used to verify the rationality and effectiveness of the registration algorithm. The cloud registration algorithm in this experiment was run on a desktop computer equipped with Ubuntu18.04 LTS, AMD Ryzen 9 5950 × 3.4ghz CPU, 64G DDR4 memory, and GTX 3070 Ti graphics card. Using Open3D (Zhou et al., 2018) and OpenCV(Bradski, 2000) and other tools and running the corresponding C++ algorithm program (Xu et al., 2021), the experimental parameters are set as follows: the maximum iteration time was set to 50, error function threshold to 10^–6^, *ϵ*_*d*_ to 0.5, *ϵ*_*σ*_ to 1.3, *ϵ*_*n*_ to 0.523, Kd-Tree search radius to 0.08, orb feature number to 500, orb pyramid layer number to 8, and orb pyramid extraction ratio to 1.2. The oriented BRIEF descriptor size was set to 31.

### 4.2 Color Information Optimization

To verify the optimization performance of the genetic algorithm for HSV color information, this article used the RawFoot as test datasets. The RawFoot database is designed specifically for the descriptor; and the classification method in robustness on light condition changes and pays special attention to the change in the light source color; the light conditions in the direction of the light, light color, and brightness on the combination of these factors are different.

**FIGURE 2:**
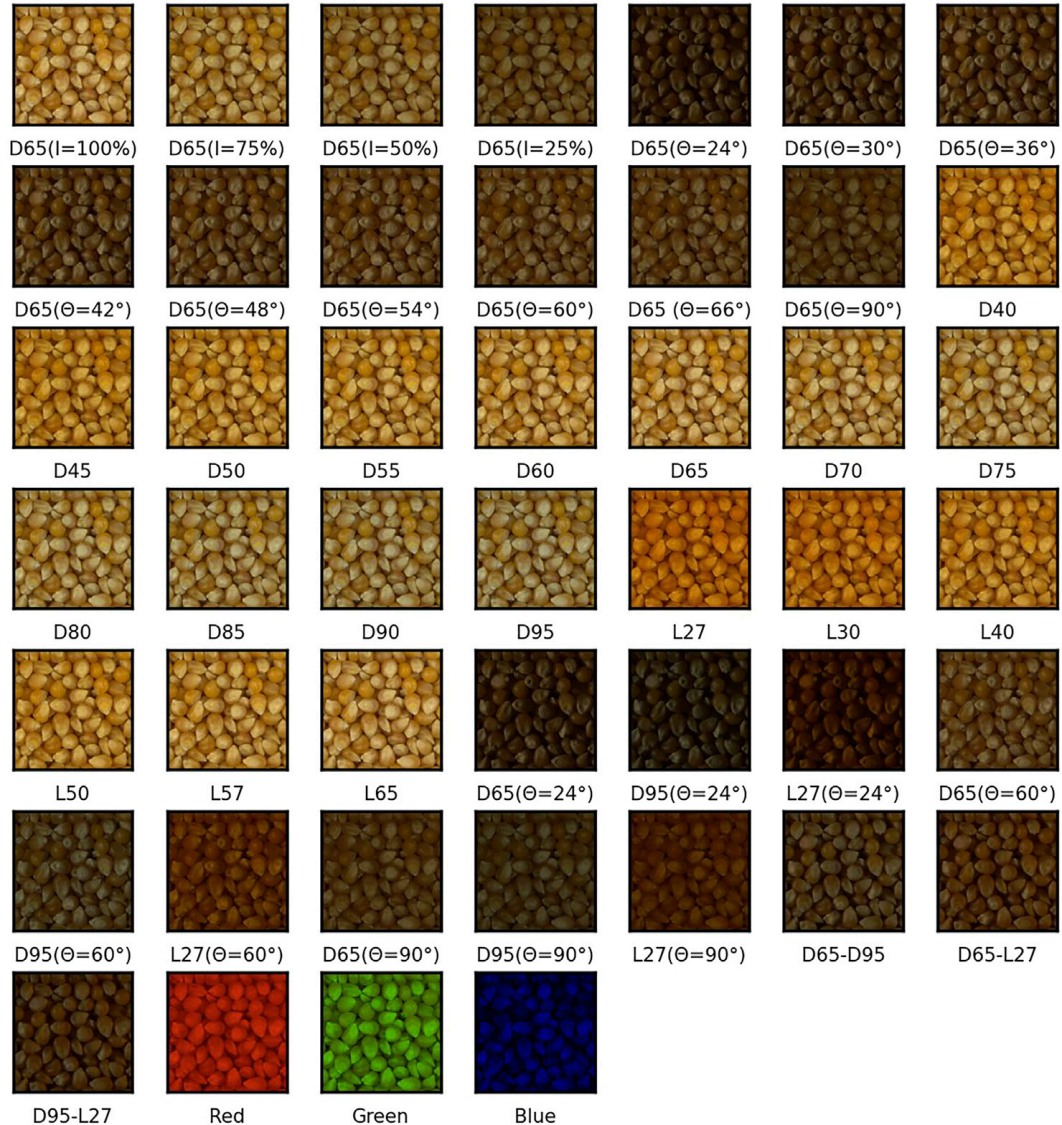
Image of the original corn sample set.

The sample set of corn is selected from RawFoot as test data. The sample set of corn is composed of 46 pictures of different light sources and angles, as shown in Figure 2. D40 is regarded as the reference image, and the original RGB, original HSV, and enhanced HSV data are compared to verify the effectiveness of image enhancement. Meanwhile, in order to effectively evaluate the consistency of the image enhancement algorithm in different lighting and light angles, the sum square distance SSD algorithm is used to calculate the degree of difference between the image under different lighting and the benchmark images:

$$\text{SSD}\left(O,E\right)=\frac{1}{N}\overset{N}{\underset{i=1}{\sum }}{\left(C\left(o_{i}\right)-C\left(e_{i}\right)\right)}^{2}.$$ (31)

*O* stands for reference pictures, on behalf of the need to compute the degree of the difference image, *o*_*i*_ and *e*_*i*_ represent *O* and *E* with a single channel of pixels, where the function represents a pixel value on a single channel of the picture. The smaller the pixel value difference between the basic picture and different lighting conditions, the closer the SSD value is. It represents the calculated value under different lighting conditions, which is closer to the original value of the reference picture.

**FIGURE 3:**
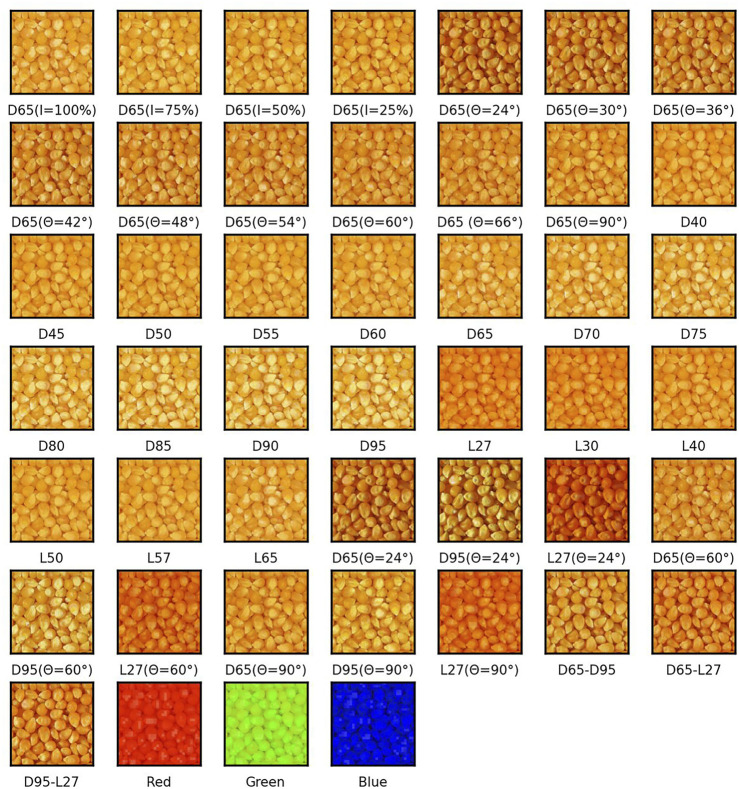
Image of the corn sample set after enhancement.

Figure 2 shows the image of the corn sample set in the original RawFoot dataset. Due to light angle and brightness, there are obvious differences between the images. Figure 3 is the result of the image enhancement algorithm. After the algorithm processing, the difference between images is significantly reduced, and the details in the dark area are more noticeable due to the improvement of brightness and contrast.

**FIGURE 4:**
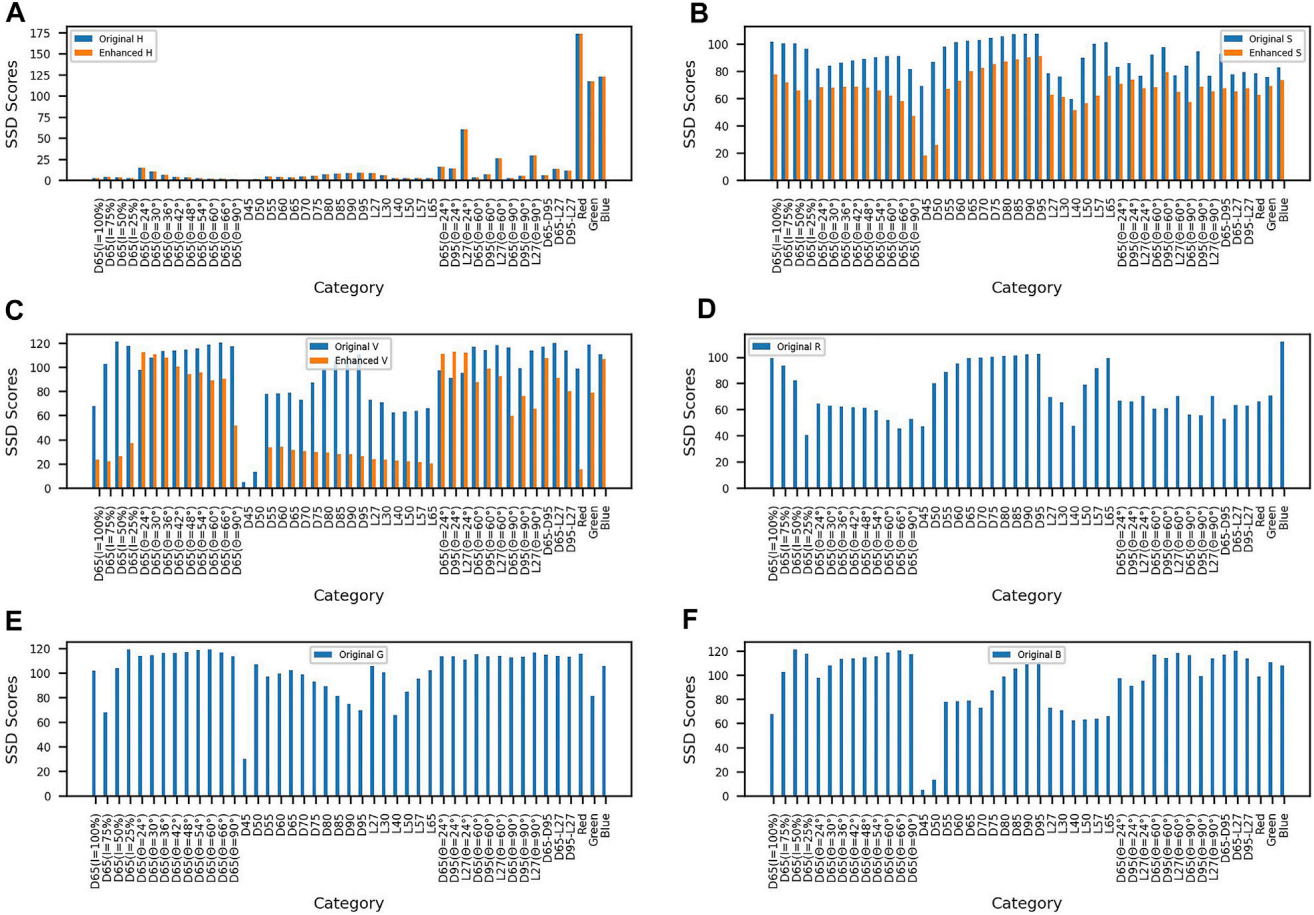
SSD contrast figure. **(A)** Comparison between the original hue and enhanced hue. **(B)** Comparison between the original saturation and enhanced saturation. **(C)** Comparison between the original value and enhanced value. **(D)** Original red channel. **(E)** Original green channel. **(F)** Original blue channel.

As shown in Figure 4, Figures 4A–C are the comparison diagrams of the original hue, saturation, and value data and the hue, saturation, and value data after enhancement; Figures 4D–F are the differences of the original R, G, and B channels, respectively. Compared with the unenhanced HSV data, the average SSD index of saturation and value after image enhancement decreased by 14.07% and 37.16% compared with the original saturation and value. Experimental results showed that the influence of illumination and light angle on saturation and value is decreased after the image enhancement algorithm.

### 4.3 Color Point Cloud Registration

The TUM dataset is a well-known RGB-D dataset, which provides rich scenes. The Kinect camera is used in the TUM dataset to collect depth information and color information, among which the resolution of the depth image and a color image is 640 * 480. In this article, rgbd_datasetet_freiburg1_xyz, rgbd_dataset_freiburg1_teddy, rgbd_dataset_freiburg1_360 and rgbd_dataset_freiburg1_plant were selected in the TUM dataset, respectively. Partial scenes were selected from four datasets of the plant for testing; ICP and NICP algorithms (Park et al., 2017) (referred to as Color 3D ICP) and the method proposed in this article were used to register overlapping color point clouds, respectively. In order to effectively evaluate other algorithms and the accuracy of the algorithm proposed in this article, we used the root mean square error (RMSE) index and the fitness index, for a given two pieces of point cloud $\left(p,\hat{p}\right)$ and $K_{p\hat{p}}$ and their matching points $\left(p_{i},{\hat{p}}_{i}\right)\in K_{p\hat{p}}$ , pit for after the rigid body transformation, including ${\left\|{p_{i}}^{t}-{\hat{p}}_{i}\right\|}_{2}^{2}$ , a representative and the distance between the ${p_{i}}^{t}$ and ${\hat{p}}_{i}$ ; RMSE metrics are defined as follows:

$$\text{RMSE}\left(p,\hat{p}\right)=\sqrt{\frac{1}{n}\overset{n}{\underset{i=1}{\sum }}{\left\|p_{i}t-{\hat{p}}_{i}\right\|}_{2}^{2}},p_{i}t=R\left(w\right)p_{i}+t_{i}.$$ (32)

In order to reflect the relationship between the number of matching points and the number of source point clouds, fitness indicators are defined as follows:

$$\text{Fitness}\left(p,K_{p\hat{p}}\right)=\frac{\left|K_{p\hat{p}}\right|}{\left|p\right|}.$$ (33)

In this article, rgbd_datasetet_freiburg1_xyz, rgbd_dataset_freiburg1_teddy, rgbd_dataset_freiburg1_360 and rgbd_dataset_freiburg1_plant were selected in the TUM dataset, respectively. Some scenes were selected from the four datasets of the plant for testing. ICP, NICP, Color 3D ICP, and the method proposed in this article were used to register the overlapping color point clouds and calculate the corresponding RMSE and fitness indicators.

Tables 1, 2, respectively, show RMSE and fitness index test results of ICP, NICP, Color 3D ICP, and our algorithm, and the best results of these algorithms are shown in bold. It can be seen from the data in Table 1 that the RMSE index of the algorithm is almost lower than that of the aforementioned algorithm, and the average RMSE is also the lowest. Meanwhile, it can be seen from Table 2 that fitness is significantly improved compared with the aforementioned algorithm, indicating that the number of feature points selected by the algorithm accounts for a large proportion of the original point cloud.

**TABLE 1:** RMSE index comparison table.

Dataset	ICP	NICP	Color 3D ICP	Our
rgbd_dataset_freiburg1_xyz	0.0225	0.0223	0.0253	**0.0185**
rgbd_dataset_freiburg1_teddy	0.0498	0.0731	0.0380	**0.0369**
rgbd_dataset_freiburg1_360	0.0469	0.0330	0.0447	**0.0326**
rgbd_dataset_freiburg1_plant	0.0483	0.0379	0.0324	**0.0321**
Average	0.0426	0.0494	0.0351	**0.0300**

Bold values represents the best result of these algorithms.

**TABLE 2:** Fitness index comparison table.

Dataset	ICP	NICP	Color 3D ICP	Our
rgbd_dataset_freiburg1_xyz	0.3804	0.3625	0.3058	**0.6742**
rgbd_dataset_freiburg1_teddy	0.2804	0.1922	0.7393	**0.7421**
rgbd_dataset_freiburg1_360	0.2967	0.3453	0.3653	**0.7121**
rgbd_dataset_freiburg1_plant	0.5116	0.3747	**0.9009**	0.9004
Average	0.3672	0.3186	0.5778	**0.7572**

Bold values represents the best result of these algorithms.

**FIGURE 5:**
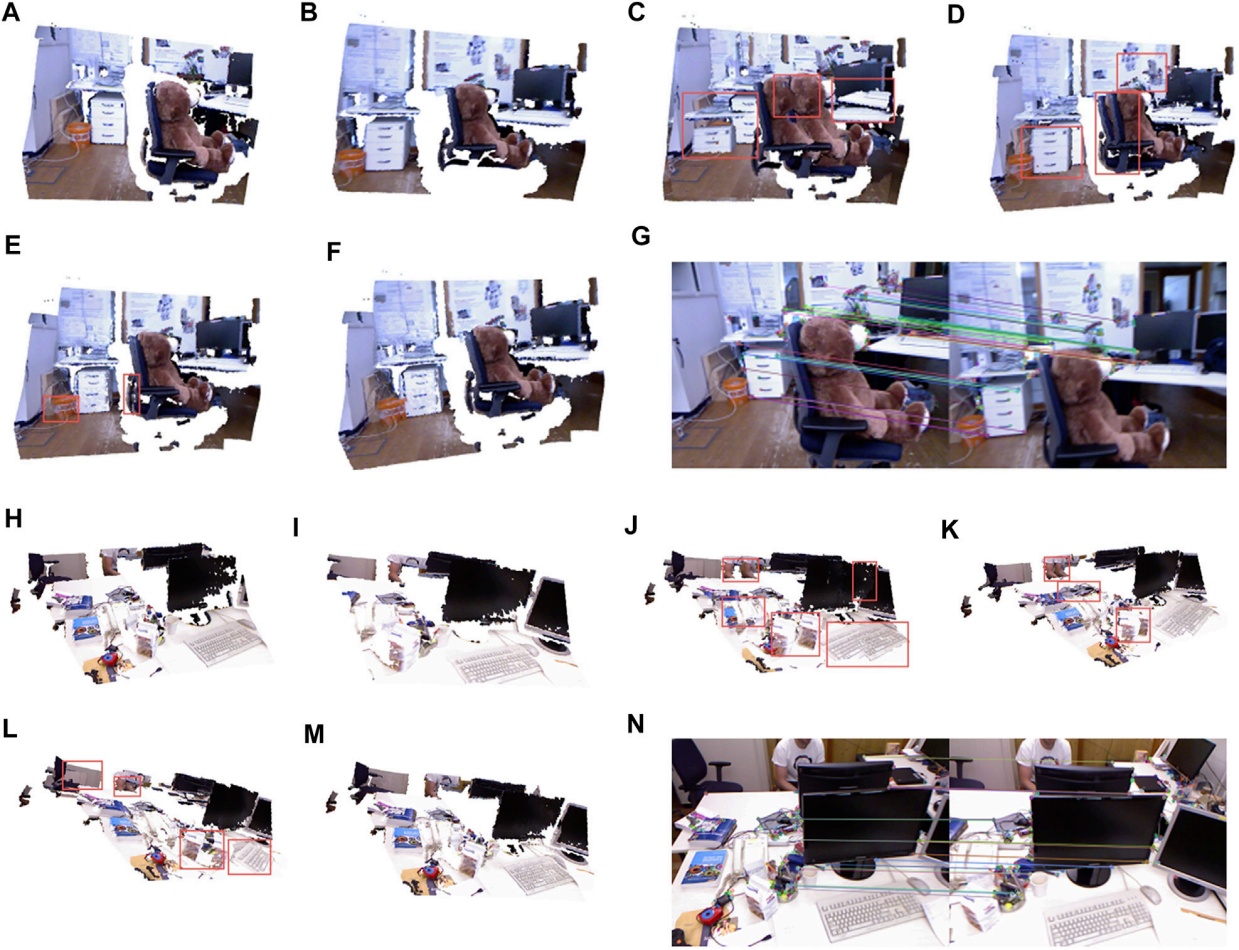
Registration results of rgbd_dataset_freiburg1_teddy and rgbd_dataset_freiburg1_xyz. **(A)** Source point cloud of rgbd_dataset_freiburg1_teddy. **(B)** Target point cloud of rgbd_dataset_freiburg1_teddy. **(C)** Result of the ICP algorithm of rgbd_dataset_freiburg1_teddy. **(D)** Result of the NICP algorithm of rgbd_dataset_freiburg1_teddy. **(E)** Result of the Color 3D ICP of rgbd_dataset_freiburg1_teddy. **(F)** Result of our algorithm. **(G)** Orb matching result of rgbd_dataset_freiburg1_teddy. **(H)** Source point cloud of rgbd_dataset_freiburg1_xyz. **(I)** Target point cloud. **(J)** Result of the ICP algorithm of rgbd_dataset_freiburg1_xyz. **(K)** Result of the NICP algorithm of rgbd_dataset_freiburg1_xyz. **(L)** Result of the Color 3D ICP of rgbd_dataset_freiburg1_xyz. **(M)** Result of our algorithm of rgbd_dataset_freiburg1_xyz. **(N)** Orb matching result of rgbd_dataset_freiburg1_xyz.

Figures 5A,B,H, and I, are the adjacent original point clouds and target point clouds randomly selected in the rgbd_dataset_freiburg1_teddy and rgbd_dataset_freiburg1_xyz data sets, respectively. Figures 5C,J are the ICP algorithm, as shown in Figure 5C. The algorithm does not fit the point cloud well and has a serious dislocation. Figures 5D,K are the NICP algorithm, which restricts the geometric structure of the point cloud by restricting the normal and curvature of the point cloud, making the algorithm better for plane registration, but the registration effect for complex objects is not ideal. Figures 5E, L show the color 3D ICP registration of point clouds by combining the color information and geometric information. However, due to the incorrect association between matching points, registration errors still exist. Figures 5F, M, N show the algorithm in this article. The algorithm in this article not only constrained the color information and geometric information but also calculated the curvatures and normals of matching points and filtered the matching points that did not meet the requirements. The results demonstrated that the algorithm in this article correctly registered the original point cloud and the target point cloud well. Figures 5G, N are orb feature point matching, and the results showed that orb feature matching for two-color images also has a good matching effect.

## 5 Conclusion

Point cloud acquisition is easy to be affected by the shooting environment. Changing the light will lead to the deviation of the brightness and color of the shooting object, which will affect the accuracy of the point cloud registration. Based on the aforementioned problems, a genetic algorithm is proposed to pre-process the color information of the point cloud. It can further eliminate the inconsistency of brightness and color caused by different lights and reduce the impact on the point cloud registration.

In this article, using the logarithmic transformation of two-dimensional image brightness and the saturation feedback formula optimized by the genetic algorithm, the algorithm is transplanted to three-dimensional point cloud illumination processing to study the sensitivity of three-dimensional point cloud registration to illumination. Its innovation lies in the combination of the saturation feedback formula and genetic algorithm to optimize the color consistency of the point cloud color information, reducing the interference of illumination factors to a certain extent.

The experiment shows that the HSV data after bionic image enhancement decrease 14.07% and 37.16% on average compared with the SSD indexes of original saturation and brightness. In addition, during the experiment, it is found that the input color image may be blurred due to jitter during camera acquisition, which will lead to blur in the registration process. Therefore, how to reduce the influence of color image blur on the accuracy of color point cloud registration is a problem that this article needs to study in the future.

## Data Availability

Publicly available datasets were analyzed in this study. These data can be found at: https://vision.in.tum.de/data/datasets/rgbd-dataset/download http://projects.ivl.disco.unimib.it/minisites/rawfoot/.
